# Infant and Child MRI: A Review of Scanning Procedures

**DOI:** 10.3389/fnins.2021.666020

**Published:** 2021-07-12

**Authors:** Anni Copeland, Eero Silver, Riikka Korja, Satu J. Lehtola, Harri Merisaari, Ekaterina Saukko, Susanne Sinisalo, Jani Saunavaara, Tuire Lähdesmäki, Riitta Parkkola, Saara Nolvi, Linnea Karlsson, Hasse Karlsson, Jetro J. Tuulari

**Affiliations:** ^1^FinnBrain Birth Cohort Study, Turku Brain and Mind Center, Department of Clinical Medicine, University of Turku, Turku, Finland; ^2^Department of Psychiatry, Turku University Hospital, University of Turku, Turku, Finland; ^3^Department of Psychology, University of Turku, Turku, Finland; ^4^Department of Radiology, Turku University Hospital, University of Turku, Turku, Finland; ^5^Department of Medical Physics, Turku University Hospital, Turku, Finland; ^6^Department of Pediatric Neurology, Turku University Hospital, University of Turku, Turku, Finland; ^7^Department of Psychology and Speech-Language Pathology, Turku Institute for Advanced Studies, University of Turku, Turku, Finland; ^8^Centre for Population Health Research, Turku University Hospital, University of Turku, Turku, Finland; ^9^Turku Collegium for Science, Medicine and Technology, University of Turku, Turku, Finland; ^10^Department of Psychiatry, University of Oxford, Oxford, United Kingdom

**Keywords:** magnetic resonance imaging, infant, child, neuroimaging, brain

## Abstract

Magnetic resonance imaging (MRI) is a safe method to examine human brain. However, a typical MR scan is very sensitive to motion, and it requires the subject to lie still during the acquisition, which is a major challenge for pediatric scans. Consequently, in a clinical setting, sedation or general anesthesia is often used. In the research setting including healthy subjects anesthetics are not recommended for ethical reasons and potential longer-term harm. Here we review the methods used to prepare a child for an MRI scan, but also on the techniques and tools used during the scanning to enable a successful scan. Additionally, we critically evaluate how studies have reported the scanning procedure and success of scanning. We searched articles based on special subject headings from PubMed and identified 86 studies using brain MRI in healthy subjects between 0 and 6 years of age. Scan preparations expectedly depended on subject’s age; infants and young children were scanned asleep after feeding and swaddling and older children were scanned awake. Comparing the efficiency of different procedures was difficult because of the heterogeneous reporting of the used methods and the success rates. Based on this review, we recommend more detailed reporting of scanning procedure to help find out which are the factors affecting the success of scanning. In the long term, this could help the research field to get high quality data, but also the clinical field to reduce the use of anesthetics. Finally, we introduce the protocol used in scanning 2 to 5-week-old infants in the FinnBrain Birth Cohort Study, and tips for calming neonates during the scans.

## Introduction

Magnetic resonance imaging (MRI) is a non-invasive and safe method to examine the human brain across the entire lifespan. Compared to the computer tomography (CT) and X-ray, MRI does not use ionizing radiation and has excellent soft-tissue contrast (Lee et al., 2017). Thus, it is well-suited also for clinical investigations carried out with pediatric population as well as for research settings including healthy subjects. However, MRI is very sensitive to motion, and therefore, the examination requires the subject to lie still during the scan. The acoustic noise of the scanner can rise up to 132 dB(A) (Foster et al., 2000), and acquisition time varies normally between 15 to 60 min, depending on the set up and amount of sequences acquired. When scanning pediatric populations for clinical purposes, moderate sedation or general anesthesia is often used to reduce anxiety and motion (Royal and Road, 2000). For ethical reasons, such as the risks related to anesthesia, sedation is not a generally accepted option for neuroimaging research examining healthy subjects (Edwards and Arthurs, 2011). However, even the participants that are not able to co-operate during the scan, can be scanned without motion during natural sleep, but this method in turn creates substantial challenges for the scan preparations. Despite these challenges, MRI plays an important role in pediatric neuroimaging research field (Zhang et al., 2019). A recent review focuses on challenges in pediatric MRI and it also covers many of the technological advances that may improve the success rate in the future (Barkovich et al., 2019).

Neuroimaging studies in healthy infants using MRI as an imaging method have increased recently, and simultaneously, the field of research has expanded to various branches of science such as psychology, logopedics, and social sciences. The human brain develops and grows in size extremely fast during the first 2 years after birth (Knickmeyer et al., 2008) and is consequently sensitive to many environmental influences (Pulli et al., 2018). To investigate what is normal development, a number of studies have been conducted in healthy individuals (Bompard et al., 2014; Li et al., 2014c; Deoni et al., 2015), at-risk populations (Hazlett et al., 2012; Dean et al., 2014b; Grewen et al., 2014; Donald et al., 2015; Langer et al., 2015; Monk et al., 2015; Ou et al., 2015; Qiu et al., 2015a, 2013b; Chang et al., 2016; Jha et al., 2016; Salzwedel et al., 2016), and clinical populations (Karmacharya et al., 2018; Moran et al., 2019), among others. To minimize postnatal environmental influences, the most common imaging time point of interest has been during infancy as close to birth as possible, generally during the first few postnatal weeks. This is surprisingly one of the most convenient time points to perform imaging as infants sleep a lot during the period right after birth (Galland et al., 2012). Nevertheless, there are various challenges starting from recruitment and timing of imaging without intruding parents’ day-to-day schedules. Further, pediatric scanning requires special expertise as these scans are seldom a routine in the clinic or in research settings.

Previously, a few publications have especially focused on scanning methods of infants and young children without sedation. The main factor affecting the selection of preparation techniques is age of the participants and developmental needs. Mathur et al. (2008) have published guidelines to perform brain MRI without sedation with neonatal intensive care unit (NICU) patients. Later on, Arthurs et al. (2012) reviewed key techniques to avoid sedation in neonatal imaging and focused on challenges like physiological changes, equipment compatibility, and acoustic noise. The key technique with neonates is feed and wrap (also termed feed and sleep, feed and swaddle, feed and bundle) and it is mainly used in infants less than 3 months old (Antonov et al., 2017). A specific vacuum fixation immobilizer is commonly used to swaddle the infant in the feed and wrap technique. Using a vacuum immobilizer is safe, low cost, and obviates the need of anesthesia (Golan et al., 2011). Questionnaire survey by Heller et al. (2017) proved retrospectively that the primary technique for conducting neonatal MRI in NICU in the United States was the feed and swaddle technique (64%), while the rest of the NICUs used primarily sedation or general anesthesia to aid the scans. The same study expectedly showed a lower success rate of quality data in the feed and swaddle group comparing to the sedation and general anesthesia groups. Further, after the first few months after birth, the feed and swaddle technique becomes more ineffective and scanning without sedation becomes more demanding. Still, Dean et al. (2014a) outlined a protocol for scanning healthy children under the age of four during natural, non-sedated sleep. In that longitudinal study, 384 MRI datasets were successfully acquired from 220 healthy subjects with an overall 97% success rate. The scans were scheduled for the evening hours, and in some cases the participants were sleep deprived. There are even more studies that have reported techniques to scan children mostly older than 4 years while awake (Raschle et al., 2009; De Bie et al., 2010). For example, Raschle et al. (2009) provided general guidelines highlighting comfort, appropriateness, and motivation (CAM). A step-by-step protocol with a video report designed for pediatric neuroimaging sessions in young children were also presented. In this age group, MRI compatible weighted blankets might be helpful to limit movement during acquisition as well (Horien et al., 2020).

Although motion prevention is carried out in the best possible way, there is always a possibility of subtle, involuntary movements during the acquisition. Even heart beats, breathing, or blinking can cause motion artifacts and reduce MRI data quality. Any kind of motion is a challenge and concerns clinical and research imaging equally. To improve the data quality, numerous methods have been developed to mitigate or correct motion (Zaitsev et al., 2015). Methods can be classified into prospective and retrospective approaches, which both contain various techniques. Prospective techniques use a real-time correction (Brown et al., 2010), while retrospective techniques modify data during the reconstruction (Loktyushin et al., 2013). Both methods have been applied in brain imaging (Godenschweger et al., 2016). However, all methods have limitations and to date, no single method can completely eliminate motion artifacts. Thus, minimization of the motion remains crucial (Reuter et al., 2015).

This review had specific goals to focus on the reporting of scanning protocols and success rates of extant studies, which has not been covered in prior reviews on the field. We dedicate a section to our own procedures that we hope will help future data collection. The first aim of this systematic review is to summarize the methods used to scan 0–6-year-old subjects in the MRI scanner focusing on the studies published during the last 9 years, with a special emphasis on procedure to prepare a child for an MRI scan, but also on the techniques and tools used during the scanning to enable a successful scan. We focused on the studies conducted on healthy, full-term subjects because the preterm born subjects are often scanned with clinical implications. Descriptions of the scanning procedures and a summary of the most commonly used techniques are reported. We also examined how the scans have succeeded, and on the other hand, considered the reasons behind the failed scans. The second aim was to provide strategies for scanning infants and young children without sedation. To increase sample sizes and the quality of data and decrease the number of drop-outs in the follow-up scans, it is important to know these methods well. In the future, these methods could even be introduced to the clinical setting as well to reduce the need of sedation during MRI. Finally, we introduce the neonatal MR protocols that were used in the FinnBrain Birth Cohort Study Neuroimaging Lab (finnbrain.fi).

## Methods

### Literature Search

The primary targets of interest were study populations consisting of term born infants and young children with focus on examining brain growth and development using MRI. A literature search using PubMed database was originally conducted on the 30th of June in 2016. The search comprised of the following keywords (‘Magnetic Resonance Imaging’[Mesh] OR ‘MR imaging^∗^’ OR ‘MRI’ OR ‘NMRI’ OR ‘fMRI’ OR ‘DTI’ OR ‘diffusion tensor imaging’) AND (‘Brain/growth and development’[Mesh] OR ‘brain growth^∗^’ OR ‘brain developm^∗^’) AND (‘Infant’[Mesh] OR ‘infant^∗^’ OR ‘toddler^∗^’). No languages were excluded at this point. To capture the most recent and relevant work in the field, a starting date limit was enforced to include only papers published after the 1st of January in 2012. The search was updated on the 9th of March in 2021 and the final search included literature published between 1st of January in 2012 to 1st of January in 2021. After duplicates were removed, the search resulted in a total of 1098 publications.

Titles and abstracts were used to screen articles in the first phase. Exclusion criteria were the following, in a descending order of priority:

(1) The publication was written in a language other than English.
(2) The study was not a human study.
(3) The study focused on the prematurely born or low birth weight subjects of any age.
(4) The study was focusing on a disease or treatment. A potential risk of carrying a disease was not a reason to be excluded as long as the disease was not detected.
(5) 0–2-year-old living subjects were not MR imaged in the study.

**FIGURE 1:**
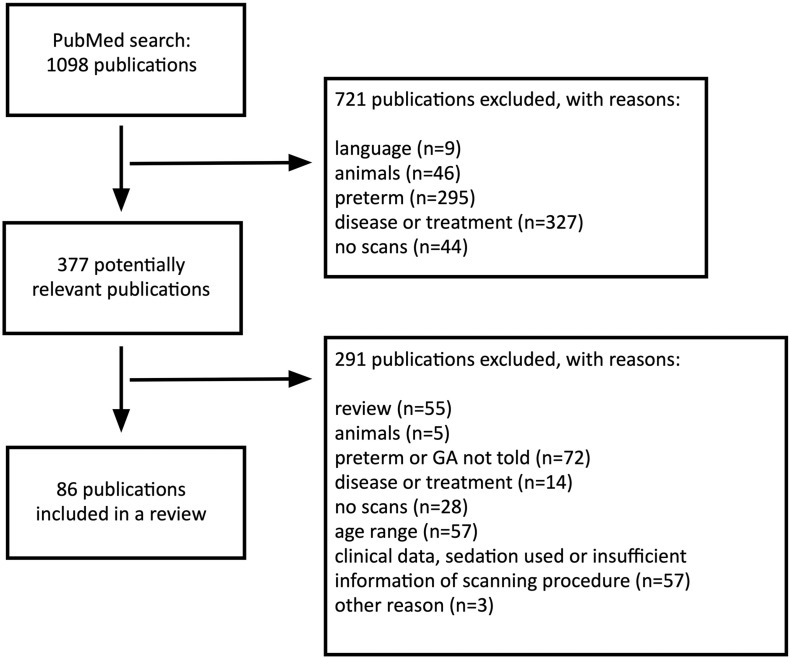
Flow diagram outlining the literature search.

If a publication met more than one criterion, only the highest criterion was marked as a reason for exclusion. If the exclusion criterion was found from the title, the abstract was not used to find a higher priority criterion for exclusion. 721 articles were screened out. 377 publications were identified as potentially relevant (Figure 1).

These 377 publications were reviewed based on the abstracts and full texts. At this second phase, we first excluded all review articles and after that, the exclusion criteria (1–5) were applied as in the first phase. Given that we were particularly interested in studies using healthy infants and young children who underwent MRI without sedation (noting that preterm-born children are frequently scanned under anesthesia), only articles that met the following criteria were included:

(1) All subjects were scanned between 0 and 6 years of age.
(2) All scans were made without sedation and MRI was not clinically indicated. To make sure no sedatives were not used, the study had to state it or mention scans were made during *natural* sleep or awake. If this was not told, publication was excluded due to insufficient information of the scanning procedure.
(3) In accordance with the study’s inclusion or exclusion criteria, only subjects born at gestational age (GA) 35 weeks or later were included. If a study set a lower limit than 35 weeks for GA, it was excluded regardless of the subjects’ GAs. If a study did not set a limit for GA and the range was not reported the mean GA was ≥ 37 weeks with standard deviation ≤ 2 weeks (and mean GA minus SD was ≥ 35 weeks). Finally, regarding studies with no mention on GA, only longitudinal studies were included.

All information was obtained from the article full-texts and their supplementary data when applicable. While we were interested in the methods to calm subjects in the scanner, we also investigated how studies have reported the used procedures before and at the scanner. Thus, reviewed studies may contain overlapping participant populations. 291 publications did not meet the criteria and were excluded.

Finally, a total of 86 original articles published in English were identified and included in this review. The included studies are listed in Supplementary Table 1. While we acknowledge that the inclusion of a longer time frame and studies with prematurely born participants would provide somewhat more information on the topic, we opted to include the most recent studies performed without clinical grounds.

## Results

### Study Characteristics

For all the included study populations (*n* = 86) MRI scans were performed on the subjects between 0 and 6 years of age. The number of participants per one study ranged from 9 to 288. Majority of the subjects were term born, but in some cases gestational age at birth was not provided. Sample sizes and ages at scan are shown in Supplementary Table 1. Fifty-two studies were completely cross-sectional and performed only one scan per each subject (Hazlett et al., 2012; Deniz Can et al., 2013; Deoni et al., 2013, 2015; Gao et al., 2013; O’Muircheartaigh et al., 2013, 2014; Qiu et al., 2013a, 2015b; Broekman et al., 2014; Dean et al., 2014b, c, 2017, 2018a,b; Grewen et al., 2014; Travis et al., 2014; Zhang et al., 2014; Donald et al., 2015; Langer et al., 2015; Ou et al., 2015; Poh et al., 2015; Spann et al., 2015a, b, 2020a,b; Ferradal et al., 2016, 2019; Li et al., 2016; Sethna et al., 2016; Adibpour et al., 2018, 2020; Lugo-Candelas et al., 2018; Monnelly et al., 2018; Chen et al., 2019; Hernandez-Castillo et al., 2019; Lebenberg et al., 2019; Lehtola et al., 2019; Ong et al., 2019; Tuulari et al., 2019; Acosta et al., 2020a, b, 2021; Alexander et al., 2020; Bruchhage et al., 2020; Camacho et al., 2020; Dowe et al., 2020; Fenchel et al., 2020; Gale-Grant et al., 2020; Graham et al., 2020; Merhar et al., 2020; Merz et al., 2020). The remaining studies (*n* = 34) were longitudinal and conducted serial scans on the same individuals (Geng et al., 2012, 2016; Choe et al., 2013; Li et al., 2013, 2014a,b,c, 2015a,b; Qiu et al., 2013b; Sadeghi et al., 2013; Alcauter et al., 2014, 2015; Bompard et al., 2014; Chen et al., 2014; Dean et al., 2014a, 2015a,b; Gao et al., 2014a, b; Swanson et al., 2015; Chang et al., 2016; Croteau-Chonka et al., 2016; Deoni et al., 2016; Kim et al., 2016; Meng et al., 2017; Ahn et al., 2019; Dai et al., 2019a, b; Hu et al., 2019; Wang et al., 2019, 2012; Remer et al., 2020; Schmied et al., 2020). Serial scans included two to seven scans per subject, most typically three scans per subject. The majority of the studies (*n* = 70) used 3 Tesla MRI scanners (Geng et al., 2012, 2016; Hazlett et al., 2012; Deoni et al., 2013, 2015; Gao et al., 2013, 2014a,b; Li et al., 2013, 2014a,b,c, 2015a,b; Sadeghi et al., 2013; Alcauter et al., 2014, 2015; Bompard et al., 2014; Chen et al., 2014, 2019; Dean et al., 2014a,b,c, 2015a,b, 2017, 2018a,b; Grewen et al., 2014; Zhang et al., 2014; Donald et al., 2015; Langer et al., 2015; Spann et al., 2015a,b, 2020a,b; Swanson et al., 2015; Chang et al., 2016; Croteau-Chonka et al., 2016; Ferradal et al., 2016, 2019; Kim et al., 2016; Meng et al., 2017; Adibpour et al., 2018, 2020; Lugo-Candelas et al., 2018; Monnelly et al., 2018; Ahn et al., 2019; Dai et al., 2019a,b; Hernandez-Castillo et al., 2019; Hu et al., 2019; Lebenberg et al., 2019; Lehtola et al., 2019; Tuulari et al., 2019; Wang et al., 2019, 2012; Acosta et al., 2020a, b, 2021; Alexander et al., 2020; Bruchhage et al., 2020; Camacho et al., 2020; Dowe et al., 2020; Fenchel et al., 2020; Gale-Grant et al., 2020; Merhar et al., 2020; Merz et al., 2020; Remer et al., 2020; Schmied et al., 2020), while 1.5 Tesla scanners were less commonly used (*n* = 12) (Choe et al., 2013; Deniz Can et al., 2013; Qiu et al., 2013a, b, 2015b; Broekman et al., 2014; Travis et al., 2014; Ou et al., 2015; Poh et al., 2015; Li et al., 2016; Sethna et al., 2016; Ong et al., 2019). Four studies did not report the field strength of the used MR scanner (O’Muircheartaigh et al., 2013, 2014; Deoni et al., 2016; Graham et al., 2020).

### Participant’s State During the Scan

All subjects underwent the MRI scanning non-sedated. Infants and children under the age of 4 slept during acquisition in majority of studies (*n* = 73/86) (Acosta et al., 2020a, b, 2021; Adibpour et al., 2018, 2020; Ahn et al., 2019; Alcauter et al., 2014, 2015; Alexander et al., 2020; Bompard et al., 2014; Broekman et al., 2014; Bruchhage et al., 2020; Camacho et al., 2020; Chang et al., 2016; Chen et al., 2019; Choe et al., 2013; Croteau-Chonka et al., 2016; Dai et al., 2019a, b; Dean et al., 2014a,b,c, 2015a,b, 2017, 2018a,b; Deniz Can et al., 2013; Deoni et al., 2013, 2015, 2016; Donald et al., 2015; Dowe et al., 2020; Ferradal et al., 2016, 2019; Gale-Grant et al., 2020; Gao et al., 2013, 2014a,b; Geng et al., 2016; Graham et al., 2020; Grewen et al., 2014; Hazlett et al., 2012; Hernandez-Castillo et al., 2019; Hu et al., 2019; Kim et al., 2016; Langer et al., 2015; Lebenberg et al., 2019; Lehtola et al., 2019; Li et al., 2014b, 2015a,b, 2016; Lugo-Candelas et al., 2018; Meng et al., 2017; Merhar et al., 2020; Merz et al., 2020; Monnelly et al., 2018; O’Muircheartaigh et al., 2013, 2014; Ong et al., 2019; Poh et al., 2015; Qiu et al., 2013a, b, 2015b; Remer et al., 2020; Sadeghi et al., 2013; Schmied et al., 2020; Sethna et al., 2016; Swanson et al., 2015; Tuulari et al., 2019; Wang et al., 2019; Zhang et al., 2014). Seven studies reported that infants were given time to fall asleep before scanning, but the subject’s state during the scan was not specifically reported (Geng et al., 2012; Chen et al., 2014; Travis et al., 2014; Spann et al., 2015a,b, 2020a,b). One study reported that infants were scanned during natural sleep or while resting quietly (Ferradal et al., 2016). Furthermore, a few studies (*n* = 6) reported scans without sedation, but subject’s state during the scan was not specifically reported (Wang et al., 2012; Li et al., 2013, 2014a,c; Ou et al., 2015; Fenchel et al., 2020). See Table 1 for a summary.

**TABLE 1:** Reporting of scanning procedures in included studies.

	*N*	Method
**Timing of the visit**	14	Scheduled for the naptime or bedtime
	72	Not reported
**Preparations at home**	3	Sleep deprivation
	3	MRI sounds
	81	Not reported
**Preparations at MRI facilities**	32	Feeding before the scan
	5	Replicating typical naptime routines
	5	MRI sounds
	1	Stimulating tasks served to fatigue
	48	Not reported
**Subject’s state during the scan**	80	Reported (sleep or awake)
	6	Not reported
**Motion prevention**	51	Immobilization (various methods used)
	35	Not reported
**Noise attenuation**	58	Ear protection (various methods used)
	20	Acquisition parameter optimization
	17	Sound insulating bore liner or foam insert placed inside of the scanner bore
	6	“Precautions”
	21	Not reported
**Monitoring during the scan**	28	Pulse oximeter
	1	Pulse socks
	26	Visually monitored
	8	Camera
	4	Electrocardiography
	47	Not reported
**Duration of the scan**	38	Reported^†^
	48	Not reported
**Total duration of the visit**	1	Reported
	85	Not reported

*^†^Something mentioned about the duration of the scan. The total duration of the scan was not required to be informed.*

### Special Notes on Scanning 4–6-Year Old Children

Fifteen studies of all the total 86 studies in this review scanned also children between the ages 4 to 6 years (O’Muircheartaigh et al., 2013, 2014; Chen et al., 2014, 2019; Dean et al., 2014a,c, 2015a,b; Deoni et al., 2015, 2016, 2013; Croteau-Chonka et al., 2016; Dai et al., 2019a, b; Remer et al., 2020). At this age, the child has typically more ability to cooperate, but in contrast to infants, it might be more difficult to get the child to fall asleep in a strange environment (see Table 2). Eleven studies reported that if tolerated by the child (in most cases ≥ 4 years old), the scan was made while the child was awake, e.g., when watching a movie or a TV show. The remaining four studies scanned during natural sleep. Otherwise preparations, motion prevention, sound attenuation, and monitoring during the scan did not differ from that of the younger children.

**TABLE 2:** Challenges at motor coordination, emotional and attention development in a different age groups.

	**Challenges at motor coordination, emotional and attention development**
**0–3 months**	• Irregular daily rhythm, fragmented sleep
	• Spontaneous movement of head, body and limbs
	• Startle response to hard/sudden noise
	• Entirely dependent on caregiver in emotional and physical regulation
	• Limited communicative abilities and underdeveloped capabilities to reflect on the surroundings
**4 months – 1 year**	• Sleep cycle maturates, longest continuous sleep during nighttime
	• Depended on caregivers in emotional and physical regulation
	• Separation anxiety
	• Close relationship with primary caregivers
	• Limited communicative abilities, receptive vocabulary starts to develop
**2–3 years**	• Sleep cycle maturates, longest continuous sleep during nighttime, no need for daytime sleep for some children
	• Rapid language development, inability to follow long instructions
	• Self-regulation capacity starts to develop (ability to regulate internal and external signals without adult’s help)
	• Testing boundaries, temper tantrums
**4–6 years**	• Characteristics and personality comes more visible
	• Better attention and self-regulation capacity (better ability to regulate internal and external signals without adults help)
	• Ability to follow long verbal instructions

### Timing of Visit

Timing of the MRI sessions were frequently scheduled according to participant’s normal sleeping/diurnal rhythm. Scanning schedules were reported in 14 studies (Choe et al., 2013; Deniz Can et al., 2013; Dean et al., 2014a, b, 2017, 2018b; Gao et al., 2014b; Travis et al., 2014; Langer et al., 2015; Lehtola et al., 2019; Tuulari et al., 2019; Bruchhage et al., 2020; Camacho et al., 2020; Dowe et al., 2020), and the remainder 72 studies did not report the timing of the visit. Imaging was frequently performed on naptimes or bedtime. In seven studies, MRI visits were scheduled for the subject’s naptime or bedtime, but the time was not specified (Gao et al., 2014b; Langer et al., 2015; Dean et al., 2017, 2018b; Bruchhage et al., 2020; Camacho et al., 2020; Dowe et al., 2020). One study scheduled visits for naptime in the late morning (Deniz Can et al., 2013) and three others in the late afternoon or early evening (Choe et al., 2013; Lehtola et al., 2019; Tuulari et al., 2019). In a few studies (*n* = 3), the majority or all the scans were performed in the evening around bedtime or at night (Dean et al., 2014a, b; Travis et al., 2014). Naptime imaging was typically used with the youngest participants, while evening hours and nighttime were typical scan times not only for infants, but also for the older participants.

### Preparations at Home

Getting ready for the upcoming MRI scan often started already at home. To habituate the infant to the scanner noise, families were provided a CD of the scanner sound, and parents were instructed to play the CD, while subjects were sleeping at home (Hazlett et al., 2012; Langer et al., 2015). In one study, mothers were given an MRI prep kit including earplugs and a portable speaker pre-loaded with MRI sounds. In this study, mothers were also encouraged to start swaddling their infant to sleep if they had not already started to do so (Camacho et al., 2020). In two studies, the child was deprived of sleep prior to scans by asking parents to wake the child up earlier in the morning or skip a nap on a research day (Geng et al., 2012; Dean et al., 2014a). To promote sleep at the imaging site, Dean et al. (2014a) reported that parents were asked to keep the child busy throughout the day before the scan. In total, 81 studies did not report preparations at home.

### Preparations at MRI Facilities

Replicating the typical bed or naptime routines in the MRI facilities was reported in five studies (Choe et al., 2013; Deniz Can et al., 2013; Dean et al., 2014a; Langer et al., 2015; Camacho et al., 2020). Parents were asked to bring along comfort items to create a homely environment at the imaging site (Langer et al., 2015). Private rooms with diaper changing and bathing facilities, rocking chairs, portacribs, blankets, soft lullaby music, and other objects were attempted to make the environment cozier (Choe et al., 2013; Deniz Can et al., 2013; Dean et al., 2014a; Langer et al., 2015). Dimmed lights at the MRI site and also around the facility, when carrying the sleeping child, were also provided (Dean et al., 2014a; Dowe et al., 2020). While creating a comfortable and homely environment, the children got to familiarize with the MRI sound simultaneously before the scanning and when they fell asleep in five studies (Langer et al., 2015; Spann et al., 2015a, b, 2020a,b). Langer et al. (2015) reported the use of interesting tasks prior to the scan to fatigue the child.

### Feeding

Feeding the child before scanning was the most commonly reported preparation (*n* = 32/86) (Geng et al., 2012; Wang et al., 2012; Gao et al., 2013, 2014a; Li et al., 2013, 2014a,c, 2016; Sadeghi et al., 2013; Alcauter et al., 2014, 2015; Bompard et al., 2014; Chen et al., 2014; Grewen et al., 2014; Zhang et al., 2014; Donald et al., 2015; Ou et al., 2015; Spann et al., 2015a, b, 2020a,b; Kim et al., 2016; Dean et al., 2017, 2018b; Ahn et al., 2019; Hernandez-Castillo et al., 2019; Lehtola et al., 2019; Tuulari et al., 2019; Alexander et al., 2020; Camacho et al., 2020; Dowe et al., 2020; Merhar et al., 2020). Sleep was promoted by adjusting a feeding schedule prior to the scan. After children were fed, they were swaddled or wrapped, or otherwise helped to fall asleep. A few studies explained this so called feed and sleep or feed and wrap method in more detail: children were fed 15–30 min prior to the scan and swaddled in warm sheets (Ou et al., 2015; Li et al., 2016).

### Motion Prevention

Swaddling or wrapping was used not only to make falling asleep easier, but also to reduce potential body movement during the scan. A number of different approaches to wrapping the infants were provided, varying from only wrapping them in sheets to placing them into an immobilizer. Several studies (*n* = 23) used specific vacuum immobilization mats, bags, or pillows to stabilize the child and reduce natural movement from breathing (Deoni et al., 2013; Zhang et al., 2014; Ou et al., 2015; Croteau-Chonka et al., 2016; Li et al., 2016; Dean et al., 2014a,b,c, 2015b, 2017, 2018a,b; Dai et al., 2019a, b; Hernandez-Castillo et al., 2019; Lehtola et al., 2019; Ong et al., 2019; Tuulari et al., 2019; Camacho et al., 2020; Dowe et al., 2020; Graham et al., 2020; Merhar et al., 2020; Remer et al., 2020). Dean et al. (2014a) reported placing a mat under the child before the child fell asleep and once asleep the immobilizer was wrapped around the child. Subject’s head was separately secured in a vacuum fixation device in eight studies (Geng et al., 2012, 2016; Grewen et al., 2014; Li et al., 2015a, b; Ahn et al., 2019; Wang et al., 2019, 2012), while only half of these also reported swaddling the child. Foam cushions, foam pads, and visco-elastic matters were also commonly used to keep the head in place and occupy the space between the subjects and the head coil. All in all, the majority of studies (*n* = 51/86) mentioned some method to stabilize the infant prior to the scan.

### Noise Attenuation

Acoustic noise levels of the MRI scanner were reduced, and the hearing of the subjects was protected with different methods. The major part of the studies (*n* = 65/86) reported the use of passive or active measures during acquisition, while 21 studies made no mention of sound attenuation. Six studies out of these 65 reporting noise attenuation mentioned taking precautions to reduce the noise, but did not delineate the methods (Qiu et al., 2013a, b, 2015b; Broekman et al., 2014; Poh et al., 2015; Ong et al., 2019). Most commonly used passive measure was ear protection: 25 studies used double or triple ear protection (Deniz Can et al., 2013; Deoni et al., 2013, 2015, 2016; Dean et al., 2014a,c, 2017, 2018a,b; Donald et al., 2015; Spann et al., 2015a, b, 2020a,b; Li et al., 2016; Monnelly et al., 2018; Dai et al., 2019a,b; Hernandez-Castillo et al., 2019; Lehtola et al., 2019; Tuulari et al., 2019; Alexander et al., 2020; Camacho et al., 2020; Dowe et al., 2020; Graham et al., 2020) and the remainder (*n* = 33) used single ear protection. For example, MiniMuffs, earplugs, headphones, sound attenuating ear protectors, electrodynamic headphones and a custom-made acoustic hood were used. In four studies, electrodynamic headphones played white noise (Dean et al., 2017, 2018a; Camacho et al., 2020; Dowe et al., 2020) and in one study soothing rain sounds (Bruchhage et al., 2020) during image acquisition. In addition to ear protection, noise levels were lessened by a noise insulating bore liner or foam insert fitted inside of the scanner bore in 17 studies (Deoni et al., 2013, 2015, 2016; Dean et al., 2014a,b,c, 2015b, 2017, 2018a,b; Croteau-Chonka et al., 2016; Dai et al., 2019a,b; Lebenberg et al., 2019; Bruchhage et al., 2020; Dowe et al., 2020; Remer et al., 2020). Furthermore, some studies reported reducing scanner noise actively by selecting specific imaging parameters, slowing the gradient switching rate and reducing the maximum gradient amplitudes (Deoni et al., 2013, 2015, 2016; O’Muircheartaigh et al., 2013, 2014; Dean et al., 2014a,b,c, 2015b, 2017, 2018a,b; Croteau-Chonka et al., 2016; Dai et al., 2019a,b; Lehtola et al., 2019; Tuulari et al., 2019; Adibpour et al., 2020; Dowe et al., 2020; Remer et al., 2020). The changes in the imaging parameters were shown to provide approximately a 35 dB noise reduction during the acquisition (Dean et al., 2014a).

### Monitoring

Numerous studies (*n* = 39/86) mentioned monitoring subjects throughout the scan. To confirm that the child remained asleep, a physician, a nurse, a research assistant or a member of the research team was presented, who visually monitored the subject in 26 studies (Geng et al., 2012; Wang et al., 2012; Qiu et al., 2013a, b, 2015b; Broekman et al., 2014; Dean et al., 2014a, 2017, 2018b; Grewen et al., 2014; Li et al., 2014b, 2015b; Donald et al., 2015; Poh et al., 2015; Chang et al., 2016; Croteau-Chonka et al., 2016; Meng et al., 2017; Monnelly et al., 2018; Hernandez-Castillo et al., 2019; Lehtola et al., 2019; Ong et al., 2019; Tuulari et al., 2019; Bruchhage et al., 2020; Camacho et al., 2020; Dowe et al., 2020; Gale-Grant et al., 2020). Three studies reported using an MRI compatible camera (Ou et al., 2015; Bruchhage et al., 2020; Graham et al., 2020), and in additional five studies an infrared camera to monitor subjects during the scan (Deoni et al., 2013; Dean et al., 2014c; Croteau-Chonka et al., 2016; Dai et al., 2019a, b). Besides visual monitoring, 28 studies used a pulse oximeter to follow the heart rate and oxygen saturation (Geng et al., 2012; Wang et al., 2012; Deoni et al., 2013; Qiu et al., 2013a, b, 2015b; Broekman et al., 2014; Dean et al., 2014a, c; Grewen et al., 2014; Li et al., 2014b, 2015b, 2016; Donald et al., 2015; Ou et al., 2015; Poh et al., 2015; Spann et al., 2015a, b, 2020a,b; Croteau-Chonka et al., 2016; Ferradal et al., 2016; Meng et al., 2017; Monnelly et al., 2018; Dai et al., 2019a, b; Hu et al., 2019; Ong et al., 2019). Bruchhage et al. (2020) reported using pulse socks to monitor pulse and behavior when asleep. Electrocardiography (ECG) was used in four studies (Spann et al., 2015a, b, 2020a,b). One study reported monitoring heart rate, oxygen saturation, and temperature, but did not specify the used equipment (Gale-Grant et al., 2020). In addition to these monitoring methods made by a professional or a member of a research team, only six studies reported parents being invited to remain in the imaging site during the acquisition (Dean et al., 2014a, 2017, 2018b; Lehtola et al., 2019; Tuulari et al., 2019; Dowe et al., 2020).

### Scan Duration

The majority of studies did not directly report the total duration of the imaging protocol. The acquisition time could often be calculated using given sequences and the number of planes, this however, does not tell the total/maximum time in the scanner. Most of the studies reported using different imaging sequences (e.g., T1, T2, DTI, fMRI), but did not report the individual acquisition times for them. When reported, the acquisition times ranged from 2 min (Chen et al., 2019) up to 2 h (for a typical MRI session) (Ferradal et al., 2016), trying to keep them short to prevent the child from waking up during the acquisition (Dean et al., 2014a). The child’s cooperation and ability to remain asleep enabled additional imaging sequences and longer imaging times (Dean et al., 2015b). In some studies, the imaging times varied depending on the subject’s age due to different protocols used in various age groups (Deoni et al., 2013, 2016; Dean et al., 2014c; O’Muircheartaigh et al., 2014; Dai et al., 2019a, b). Finally, only one study reported the duration of the whole scanning visit (Dean et al., 2014a). In this study, the duration of the visit was highly variable from less than 1 h to more than 5 h.

### Success Rate and Missing Scans

All 86 studies had successful MRI scans and the number of included scans varied between 9 (Zhang et al., 2014) to 445 (Dai et al., 2019b). In addition to the successful scans, reporting about data losses it varied considerably between studies. 41 studies (*n* = 41/86) reported the number of excluded scans or alternatively the success rate for included data (Hazlett et al., 2012; Deniz Can et al., 2013; Qiu et al., 2013a, b, 2015b; Sadeghi et al., 2013; Bompard et al., 2014; Broekman et al., 2014; Dean et al., 2014a, b, 2017, 2018a,b; Li et al., 2014c, 2016; Travis et al., 2014; Alcauter et al., 2015; Donald et al., 2015; Langer et al., 2015; Poh et al., 2015; Spann et al., 2015a, 2020b; Chang et al., 2016; Ferradal et al., 2016, 2019; Sethna et al., 2016; Adibpour et al., 2018, 2020; Lugo-Candelas et al., 2018; Dai et al., 2019a; Ong et al., 2019; Tuulari et al., 2019; Acosta et al., 2020a,b, 2021; Bruchhage et al., 2020; Camacho et al., 2020; Dowe et al., 2020; Fenchel et al., 2020; Graham et al., 2020; Merz et al., 2020). Additionally, some studies mentioned reasons for exclusion during the data preprocessing steps but did not report the number of excluded scans. Remainder of the studies used only subjects with successful scans or for some other reason did not report the number of missing scans. The number of included and excluded scans, and if available, reasons for exclusion are shown in Supplementary Table 1. The most common specified reason for losing data was movement during the scan causing motion artifacts to the data. Other mentioned reasons related to the scanning procedure were that the subject did not fall asleep prior to scanning, woke up during transition to the scanning bed or during the MRI acquisition. Exclusions were made also due to demographic reasons, problems with the analysis, age under or over the study-specific range, missed measurements in other parts of the study, and due to a brain anatomical anomaly. In other words, not all exclusion criteria were a consequence of failure at imaging. Due to the different reasons of exclusion, the success rates between studies are not comparable and do not represent exclusively the success of scanning. Instead of the overall success rate, some studies reported separate rates for different parts of the sample, for example, for age groups or for cases and controls separately. For example, Li et al. (2014c) used data acquired at three time points with following success rates: 90% for neonates, 66% for 1-year-olds, and 60% for 2-year-olds. Dean et al. (2014a) observed that scanning during the second or third visit succeeded more often (success rate 100%) than scanning during the first visit (success rate 90%).

### The Infant Scanning Procedures of the FinnBrain Study and How to Calm Infants in the Scanner

We refer to FinnBrain infant MRI measurements in the current article. They were carried out in accordance with the Declaration of Helsinki, and the protocol was approved by the joint Ethics Committee of the University of Turku and the Hospital District of Southwest Finland.

In the FinnBrain Birth Cohort Study, 189 families participated in the scans when their infants were 2–5-weeks old (*M* = 26.04, SD = 7.6, range = 8–51 days corrected for gestation), and 180 were scanned. The data collection took place between 2012 and 2016. Most scans were conducted from the afternoon to early evening hours (16:30–20:00), but ca. 10 scans took place on Saturday afternoons. The imaging was performed at the Department of Radiology, Turku University Hospital, Finland. All scans were obtained using a Siemens Magnetom Verio 3T scanner (Siemens Medical Solutions, Erlangen, Germany).

At the start of the visit, the families were welcomed by a trained and experienced radiographer and the researchers at the scanning site. The infants were fed with breastmilk or formula and swaddled into a vacuum mattress. No sedatives were used. Deformable wax plugs and custom-sized earmuffs were used for hearing protection. Parents were provided with standard earmuffs, as they stayed in the scanning room during the whole scanning session. The personnel observed the scanning from the control room through a window with a microphone contact to a parent. A loudspeaker sent the sounds from the scanning room to the control room allowing the staff to hear if the infant woke up. The session was ended if the infant did not fall asleep before or did not fall back asleep during the scan.

**FIGURE 2:**
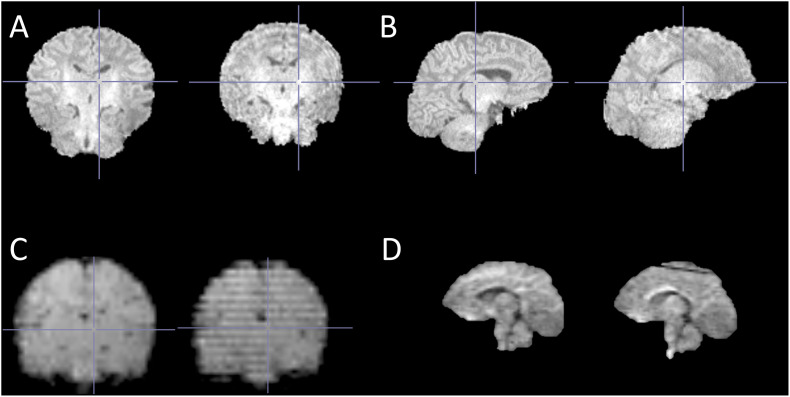
Representative examples of successful and unsuccessful neonate MRI scans (randomly selected from our data). **(A)** T1-weighted structural image with no motion artifacts (left) and with typical “ringing” motion artifact (right); **(B)** same images as in A in sagittal view; **(C)** fMRI images with no motion artifacts (left) and with typical “striping” motion artifact (right); and **(D)** diffusion-weighted image with no motion artifacts (left) and a typical “loss of signal” artifact at the superior part of the image (right). Of note, these examples are not exhaustive and are provided for visualization purposes only.

The sequences comprised of an axial PD-T2-TSE (Dual-Echo Turbo Spin Echo), a sagittal 3D-T1 (T1-weighted MPRAGE) and three diffusion tensor imaging (DTI) sequences respectively. The acquisition times for sequences were 6 min 50 s (axial PD-T2-TSE), 4 min 3 s (3D-T1), 5 min 3 s (DTI 1), 5 min 33 s (DTI 2) and 5 min 42 s (DTI 3). Sequence parameters were optimized so that “whisper” gradient mode could be used in PD-T2 TSE and 3D-T1 sequences to reduce acoustic noise during the scan (Lehtola et al., 2019). Functional MRI sequences were added to the protocol starting in June 2015 and performed until the end of the study (*N* = 28). We acquired task fMRI measurements investigating touch responses and a resting state fMRI scan. 60 min was the maximum duration of the complete scanning protocol, and total duration of the visit was less than 2 h. The data are still being processed and analyzed, but the current success rates are 125/180 for structural T1 and T2 scans (69%) (Acosta et al., 2020a), 172/180 (95%) for at least 20 good quality diffusion weighted images out of the acquired 96 images (*N* = 157 for 30 directions, *N* = 142 for 40 directions and *N* = 121 for 60 directions) (Merisaari et al., 2019). Success rates for task fMRI are 10/13 (77%) in preliminary findings (Tuulari et al., 2019) and 21/28 (75%) for the resting state data (Rajasilta et al., 2020). Unfortunately, we suffered from some technical difficulties with the T2-weighted images that were identified only after data collection, which significantly impacted the available good data from 92% estimated at the scanner to 69% in the final data. Figure 2 shows representative examples of successful and unsuccessful neonate MRI scans (randomly selected from our data).

**FIGURE 3:**
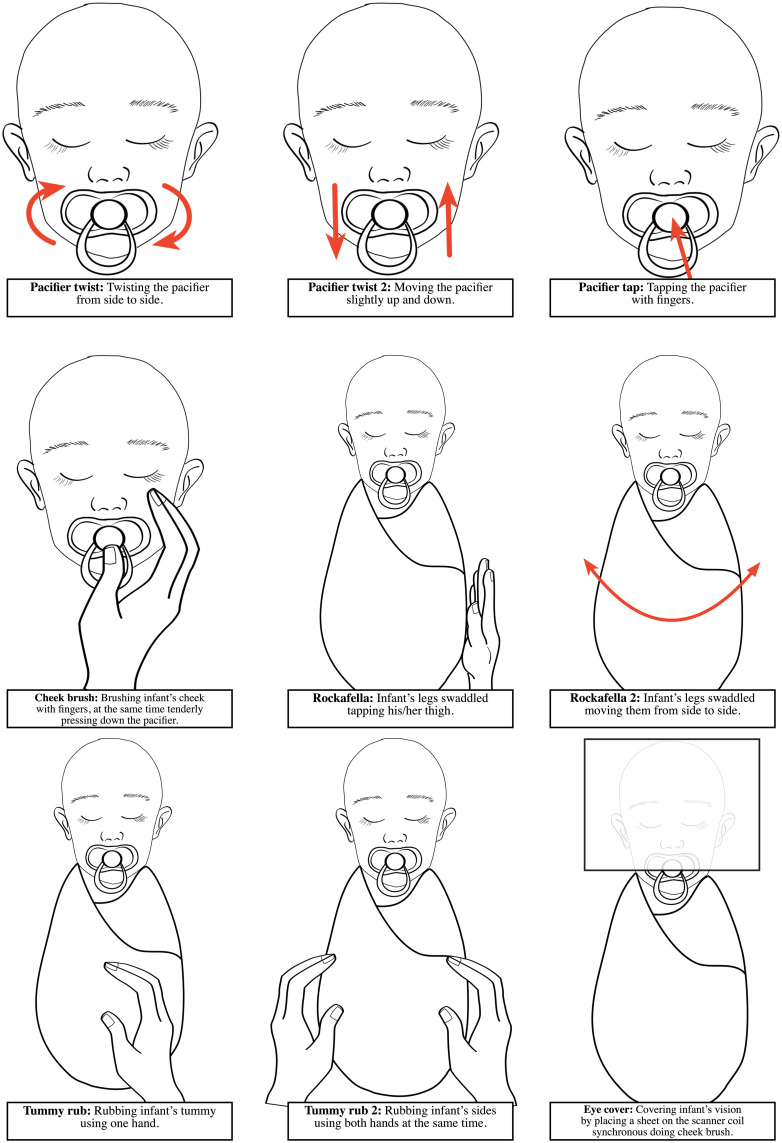
Schematic illustrations of the procedures used to calm the infants if they woke up during the scan in the FinnBrain Study. All procedures can be made by carefully reaching inside the scanner bore.

To share practical advice, we also report some procedures that we used to calm the infants if they woke up during the scan, and that can be used by gently reaching for the infant even within the scanner bore. These procedures are presented in Figure 3. During the scans, either the investigators or the parents frequently calmed the infants with these relatively simple measures that rely on infant reflexes and/or calming touch. Of important note, the homogeneity of the static magnetic field may be affected when an adult reaches into the scanner bore. Usually, we used the soothing technique for a brief period of time if the baby was sleeping restlessly at the start of the session or started to move during scanning. We let the ongoing sequence to continue during the soothing (as abrupt changes in the surrounding noises risk waking up the infant), but always acquired the sequence again, i.e., the sequences during which soothing was used were considered failed and were never used in analyses. We did not record the total number of infants who woke up during the scan, were successfully calmed and how long it took for babies to be calmed and able to put back into the MRI scanner. This would be valuable data to collect in future studies.

## Discussion

We systematically identified 86 studies, which performed infant or child brain MRI without sedation or general anesthesia in 0–6-year-olds. The majority of the studies acquired MRI scans only in subjects 2 years or younger. In this review, we concentrated on methods used to prepare a child for an MRI scan, but also on the techniques and tools used during the scanning to enable a successful scan. The most commonly used preparations especially with younger participants were feeding and wrapping the child just before the scan. This so-called feed and wrap or feed and swaddle method is widely accepted and used also in infants undergoing MRI for a clinical purpose. Also a recently published manuscript with a focus on performing pediatric neuro MRI suggested feed and swaddle to be the first-line method for subjects 3 months and younger (Barkovich et al., 2018). The efficacy of the technique has been evaluated and it has got a high rate of success (79% answered the clinical question, 20% partially answered the clinical question) in infants 3 months or younger (Antonov et al., 2017). Unfortunately, this fairly easily applicable method is less useful with older children, who do not easily fall asleep in an unfamiliar environment.

It is suggested that children from 1 to 5 years of age might be the most challenging target group to scan without sedation (Barkovich et al., 2018). Further, there is less research in this age group as compared to younger or older age groups. One practical method is to schedule the MRI visit during child’s nap or bedtime and scan during natural sleep, yet it requires flexible scanning schedules and might take time to get the child to fall asleep. Another possibility is to scan while the child is awake. However, there is no clear evidence for the best age for starting to scan the children when they are awake. Obviously, this depends on the individual child. Actually, as young as 2-year-old children have been successfully scanned awake using progressive behavioral training method (Vannest et al., 2014). This training procedure was limited to 15 min and contained 3 steps: first children were asked to sit on the scanner bed, then to lie down, and then to hold “still as a statue”. Stickers were given as reinforcers at each step. After these preparations 95% children at the age of 2–6 years had at least one successful scan sequence. Another used preparation method is to simulate the real MRI experience with mock scanner before scanning (Carter et al., 2010). This method requires language and cognition skills and is usually used with older children. However, Thieba et al. (2018) have applied mock scanner training already as young as 2-year-old children, but surprisingly, the scans with children aged 2–5 years were no more likely to be successful after receiving the mock scanner training than the ones without the use of mock scanner, although the image quality was slightly higher in the former. When the child comes more able to cooperate, capable of moving, and expressing their own will, the imaging while awake comes easier to implement.

Based on the reviewed studies, the age of 4 years seems to be the most common age to start scanning when the child is awake. In this age group, the preparation methods, including a mock scanner and behavioral training, become common in preparing children to MRI. In addition to these highly known methods, a newly emerging technology using Virtual Reality (VR) has opened up a new approach to prepare children for MRI (Ashmore et al., 2019). When applying a training protocol with a mock scanner, a success rate of 88% (53/60) for structural and 64% (23/36) for functional MRI has been obtained in a group of 4–7-year-old children (De Bie et al., 2010). Similarly, Cavarocchi et al. (2018) have reported a 83% (162/195) success rate, and an overall 30% decrease in the need of sedation after the training protocol with a mock scanner in a large cohort of pediatric patients aged 4 to 14 years. It appears that the mock scanner is most effective in children between ages 3 to 8 years (Carter et al., 2010). Unfortunately, mock scanners are rather expensive, which limits their availability. However, there is evidence that using a cheap play tunnel simulating the MRI environment might be a useful alternative (Barnea-Goraly et al., 2014). Theys et al. (2014) have developed a behavioral training protocol termed the ‘submarine protocol’ to prepare children for scanning. After completing the required tasks that made the child more familiar with potentially difficult aspects of MRI, she/he was ready for the ‘submarine ride’. The method has been used to acquire advanced MRI techniques (DTI, fMRI) in 5- and 6-year-old typically developing children with a success rate of 95% (72/76) for completing the full 35-min scan. Recently, 95% success rate in children aged 4–6 was obtained using multi-faceted concepts including an interactive app, a trained pediatric team, a children’s lounge with a toy scanner and a child-friendly multimedia environment in the MRI room (Runge et al., 2018). Surprisingly, meta-analysis demonstrating the efficacy of pre-MRI training (including booklet, audio, video, toy model, or a mock scanner) did not improve data quality, sedation use or success rate of scanning (Li et al., 2019). Authors thought that one possible account might be that training increases anxiety and fear among children. However, there were a limited number of studies (*n* = 5) and small sample sizes, thus more studies are needed to confirm the findings. All in all, success rates between studies are not readily comparable, therefore a controlled study investigating the actual effects of different methods should be performed in the future. Importantly, there is also lack of objective standard criteria of what is considered good quality data, and to what extent it can be improved with post processing.

To improve comfort and ensure safety during acquisition, noise attenuation during the scan must be executed properly. This review suggested that the most commonly employed methods were ear protection using earplugs (wax, foam or silicon), soft shell earmuffs, regular ear protectors or combinations of these. Sound-insulating bore liners or foam inserts were less frequently used. The combination of methods have been demonstrated to be more effective than one method individually (Tocchio et al., 2015). To assure sufficient noise reduction during MRI scanning in newborns, Nordell et al. (2009) have suggested the combination of dental putty fitted into the outer ear canal, earmuffs placed over both ears, and acoustic hood of dampening material placed over the child. However, passive noise control methods suffer from limitations such as discomfort, fitting problems, and very importantly, in some cases, insufficient noise reduction. Therefore, active methods like quiet sequences and quiet coils have been developed to perform scanning more quietly (McJury and Shellock, 2000). In contrast to methods trying to reduce or eliminate the acoustic noise, a new acquisition method (MR Fingerprinting-Music) has been developed to make the sounds more pleasant by emulating music (Ma et al., 2016). To our knowledge, there are no studies showing how the method works with children.

There is a general awareness that motion during acquisition causes image artifacts, which can lead to unusable data. Based on the studies reviewed here, motion was the most common reason for data exclusion. However, the systematic effects of motion are poorly known, and often uncontrolled for. This can be problematic especially in research settings, where factors of interest like age, sex, or disease are usually correlated with both the amount of head motion and structural changes. For example, head motion during MRI acquisition has been shown to influence estimates of gray matter volume and thickness (Reuter et al., 2015). Consistent with previous studies and this review, swaddling or wrapping are the most commonly used techniques to restrict motion during infant scanning. These methods are simple, low cost, and easily available but unfortunately useless in older age groups. Additionally, special vacuum immobilizer mats, cushions, and foam pads were used to stabilize body and head position. Older children are often able to watch movies or TV shows during acquisition, which has been shown to reduce the head movement. Recently, Greene et al. (2018) have developed this further by testing movies and real-time visual head motion feedback simultaneously. Both methods significantly reduced movement, but interestingly, no compounding effect of combining methods was found. Additionally, these methods can be problematic during fMRI imaging, due to the possible effects on functional data (Greene et al., 2018). To improve compliance and minimize cognitive load during functional imaging, a movie paradigm, *Inscapes*, have been conducted (Vanderwal et al., 2015). However, physical head restraint methods, videos, or behavioral strategies do not completely eliminate motion, thus motion correction later on is needed in any case. These techniques become even more crucial with high field MRI systems, such as 7 Tesla scanners, which can generate higher levels of acoustic noise, require longer acquisition times, and are more sensitive to motion artifacts (Stucht et al., 2015; Keuken et al., 2018).

In addition to preparation methods, individual factors like subject’s age, sex, culture, medical history, behavioral characteristics, and parental expectations might have an effect to MRI procedure compliance. Herein reviewed studies did not specify individual factors associated with successful scanning. Though, some studies reported separate success rates for different groups of the sample, for example, for age groups, for sexes or for cases and controls separately. However, these rates did not provide reliable information about the effect of individual variables on the compliance of MRI in children. Previously Cahoon and Davison (2014) have found that parental expectations and ratings of how well the child normally handles medical procedures were the strongest predictors of MRI compliance, while child attention problems and poor adaptability among children related to non-compliance. Thieba et al. (2018) have shown that higher cognitive and language ability in children may predict success. Finally, there is an evidence that child’s temperament may have an effect on ability to undergo MRI without sedation (Voepel-Lewis et al., 2000). These subject specific factors can cause unwanted sample selection during different phases of a study (e.g., during recruitment, preparations and imaging) and later on have an effect on interpretation of the results. To minimize these kinds of effects, a child’s MRI compliance could be determined beforehand, which might facilitate the preparation protocol. In the future, this kind of approach might be helpful. Finally, it is important to note that compliance may be related to e.g., temperament features, developmental stage, and severity of symptoms on clinical populations and may thus cause bias in samples.

All in all, a lot could be done to enhance the success rates of scans and data quality without any concrete devices, by merely paying attention to the environment, atmosphere, and suitable communication with the child and parents before, during, and after scanning. The main goal is to diminish anxiety and distress by creating a comfortable and child-friendly environment. It is essential to take into account the child’s individual and developmental needs (see Table 2) and tailor preparations accordingly. Parent comfort is equally important, because it influences directly the child’s feelings. To improve the communication with the child and family, a child life specialist (CLS) can be used in the front-line interaction. For example, parent and staff satisfaction as well as child pain and distress have been shown to be positively impacted by the child life services in pediatric imaging (Tyson et al., 2014). Finally, positive feedback and thanking the parents for their participation are needed regardless of the scanning success. It is essential to involve the child and the family in a positive overall experience, in any case, but especially if follow-up scans are under consideration. Collecting the feedback from parents, and if available from children, may help to improve protocols in the future.

MRI methods are constantly developing (Börnert and Norris, 2020). New techniques such as compressed sensing (Lustig et al., 2007), simultaneous multi-slice imaging (Larkman et al., 2001; Breuer et al., 2005) or recently developed image reconstruction algorithms utilizing artificial intelligence can reduce total scan duration significantly. For example, in the case of the protocol from FinnBrain study, duration of PD-T2 TSE and DTI sequences could easily be reduced by at least 12 min just by using multi-slice imaging. It is likely that reduced scan time would lead to better success rate of the scans.

Finally, the methods described in this review are not specific to any research setting and may equally be applied clinically. A well-known benefit of scanning without a pharmacological intervention is its safety. Many critically ill and especially chronically ill children are exposed to series of MRIs. Reducing the need of expensive and risky anesthesia would benefit these patients and the health system. Furthermore, scanning without anesthesia is cost effective (Runge et al., 2018), reduces the workload of the anesthesiology department, and may shorten the total time of visit (Vanderby et al., 2010).

### Suggestion for Reporting Scan Procedures

After conducting this review, we suggest that all studies using MRI in infant or child studies should report at least the following points about the procedure: (1) timing of visit, (2) preparations at home and MRI facilities, (3) subject’s state during scanning (asleep/awake), (4) motion prevention techniques, (5) sound attenuation techniques, (6) monitoring during scanning, (7) scan duration, (8) exact number of included and excluded scans, (9) reasons for lost data, and (10) total duration of visit.

### Limitations

This review has its limitations. First, although our literature search was wide, we might have missed articles that were not identified with our keywords (subject to MESH terms). Second, our strict selection criteria set limits especially for scanning children at the older ages. We included only studies, which scanned children at the age of 6 or younger but which also included also at least one subject under the age of 2. Because of this, the review does not give a real view of the methods and challenges in performing MRI with older children and this topic warrants thorough treatment of its own. Reviewed studies did not apply methods like a mock scanner or play therapy probably because the majority of the subjects were still too young to take advantage of them. All in all, our main interest was scanning during the first few years of life, thus the presented methods are best applied in this age group. Third, we did not exclude studies using the same or overlapping sample (the review was performed study-by-study), which can be seen in the frequency of used methods. Fourth, in this review we also highlighted the difficulties of the imaging procedure and listed the reasons for lost data, which critically viewed is reported poorly. Various reasons leading to data exclusion made it difficult to classify reasons for failure and to perform a reliable synthesis about success rates between imaging techniques, e.g., structural MRI vs. diffusion or functional MRI. In addition, some studies identified retrospectively only subjects with good quality data, so the detailed information of success rates was missing. Due to heterogeneity of the reporting, the success rates were not comparable between studies. We were open to conduct meta-analysis over the success rates, reported procedures, and timing of the studies but unfortunately this was not possible due to the high variability in reporting. Finally, we did not cover prospective and retrospective motion correction techniques or best practices of determining the degree of tolerable motion, which deserve a thorough treatment of their own.

## Conclusion

In conclusion, performing brain MRI in infants and young children without sedation is challenging, but when well-prepared, feasible to implement. This review demonstrates that there are various approaches to prepare a child for scanning, take care of noise attenuation and motion restriction. Finally, this review shows that the scanning procedures are often inadequately reported. To find out the best preparation methods and improve the success rates of scanning, we recommend reporting the procedure in more detail. In the long term, it may be possible to translate the best scan practices to the clinical field and reduce the need for anesthesia in pediatric neuroimaging (in non-urgent settings).

## Author Contributions

AC and JT conceptualized the study. The original draft was written by AC and reviewed and edited by ESi, RK, SL, HM, ESa, SS, JS, TL, RP, SN, LK, HK and JT. Supervision was provided by JT. Funding was provided by HK and JT. All authors provided critical feedback, helped shape the manuscript and accepted the final version of manuscript.

## Conflict of Interest

The authors declare that the research was conducted in the absence of any commercial or financial relationships that could be construed as a potential conflict of interest.
