# Supplementary material for: Infant and Child MRI: A Review of Scanning Procedures
Source: Front Neurosci. 2021 Jul 12;15:666020. doi: 10.3389/fnins.2021.666020 (PMC8311184; doi:10.3389/fnins.2021.666020)
Supplement: Supplementary file 1 [file Table_1.docx]

Table 1. Characteristics of included studies.

| **Study** | **Sample** | **Age at scan, mean (SD) [range]** | **Successful scans / total attempted scans if told** | **Number of excluded scans and reasons for exclusion** |
| --- | --- | --- | --- | --- |
| **Cross-sectional studies, participant age 0-3 months** | | | | |
| (Hernandez-Castillo et al., 2019) | N=25 | neonates | 25 | Not reported. |
| (Ferradal et al., 2016) | N=14 | 2–3 days | 9/14 | n=2 excessive motion during MRI  n=3 excessive motion during DOT, thus also MRIs were excluded |
| (Zhang et al., 2014) | N=9 | 2-13 days, PMA 39­–42.8 weeks | 9 | Not reported. |
| (Monnelly et al., 2018) | N=40 (n=20 prenatal methadone-exposed, n=20 non-exposed) | exposed: 3 [1-21] days  non-exposed: 13 [5-29] days | 40 | Not reported. |
| (Li et al., 2016) | N=34 (n=16 born to obese mother, n=18 born to normal weight mother) | 2 weeks  Obese: 14.3 (1.7) days  Normal weight: 14.2 (1.8) days | 34/44 | n=4 invalid structural MRI scan  n=5 due to excessive motion during the PR-fMRI scan  n=1 due to incomplete clinical data |
| (Ou et al., 2015) | N=28 (n=11 born to obese mother, n=17 born to normal weight mother,) | 2 weeks  Obese: 2.2 (0.5) weeks  Normal weight: 2.1 (0.2) weeks | 28 | Not reported. |
| (Poh et al., 2015) | N=91 | 5-17 days, PMA 38.63 (1.21) weeks | 91/189 | n=47 no DTI data  n=20 poor DTI data quality  n=7 demographic reasons  n=24 problems with the analysis |
| (Broekman et al., 2014) | N=93 | 5–17 days, 9.9 (2.3) days | 93/189 | n=13 neonates with gestational age at birth less than 37 weeks,  n=11 with birth weight less than 2500 g  n=2 with a 5-min Apgar score less than 9  n=52 with no DTI data  infants whose mothers had gestational diabetes (n=22), hypertension (n=5), and hypoglycemia (n=1) during pregnancy mothers who reported consuming any alcohol during pregnancy (n=3) ^‡^ |
| (Qiu et al., 2015b) | N=146 | 5-17 days | 146/189 | n=5 large motion on T2-weighted MRI  n=4 with no maternal anxiety measure  n=8 with no genetic data  n=26 due to demographic reasons |
| (Qiu et al., 2013a) | N=161 | 5–17 days | 161/189 (T2-weighted MRI)  110/124 (DTI) | n=18 due to demographic reasons  n=10 with large motion in the MRI |
| (Ferradal et al., 2019) | N=20 | 24.2 (7.9) days | 20/49 | n=20 due to incomplete data  n=9 due to poor data quality |
| (Donald et al., 2015) | N=73 (n=28 alcohol exposed, n=45 healthy controls) | 2-4 weeks  Alcohol exposed: 20.54 (5.98) days  Healthy controls: 22.24 (6.11) days | Alcohol exposed: 27 or 28^‡^/62  Healthy controls: 45/85  Total: 73/147 | n=44 poor spatial normalization to the infant template,  n=30 missing T2 data |
| (Dean et al., 2018b) | N=143 | 1 month, 34.1 (7.7) days^†^ | 143/149 | n=6 due to unusable imaging data (did not fall asleep, woke up during acquisition or data were corrupted by motion artifacts) |
| (Dean et al., 2018a) | N=101 | 1 month, (33.07) [18-50] days | 101/149 | n=46 due to inability to sleep through the entire diffusion scan  n=2 due to mothers did not complete all measures |
| (Dean et al., 2017) | N=104 | 1 month, 32.7 (5.81) days^†^ | 104/149^‡^ | n=33 woke up prior to and  n=13 woke up during the diffusion acquisition |
| (Lugo-Candelas et al., 2018) | N=98 (n=16 with in utero SSRI exposure (SSRI), n=21 with in utero untreated maternal depression exposure (PMD), n=61 healthy controls (HC)) | SSRI: 4.29 (1.81) weeks  PMD: 3.03 (1.65) weeks  HC: 3.30 (1.27) weeks | 98/103 | n=5 due to excessive head motion |
| (Lehtola et al., 2019) | N=68 | 2-5 weeks, 25.5 (7.8) days | 68 | Not reported. |
| (Tuulari et al., 2019) | N=10 | 2-5 weeks, (25.0) [13-31] days | 10/13 | n=3 due to excessive motion |
| (Spann et al., 2015b) | N=37 (n=24 exposed to regional anesthesia, n=13 unexposed) | 0-6 weeks  Exposed: PMA 42.3 (2.1) [36.1-45.7] weeks  Unexposed: PMA 41.7 (1.5) [40.1-45.1] weeks | 37 | Not reported. |
| (Spann et al., 2015a) | N=33 | 0–6 weeks, PMA 42.0 (1.9) weeks | 33/48 | n=11 unusable anatomical MRI data  n=4 with missing Bayley-III data |
| (Grewen et al., 2014) | N=119 (n=33 cocaine-exposed infants with or without in utero exposure to marijuana, alcohol, nicotine, opiates and/or SSRIs (PCE); n=40 infants with in utero exposure to these same substances but without cocaine (NCOC); n=46 drug-free controls (CTL) | 2–6 weeks  PCE: PMA 307.5 (2.9) days  NCOC: PMA 304.8 (2.7) days  CTL: PMA 303.1 (2.5) days | 119 | Not reported. |
| (Merz et al., 2020) | N=41 | 2.7 (1.3) [0.5–6.6] weeks | 82 (MRI)  41 (MPCSI) | N=154 were offered newborn MRI scans. Of these 154, 82 underwent MRI scans.  Reasons for not acquiring newborn MRI data were the following:  n = 18 withdrew from the study,  n = 6 declined MRI scan during the pilot portion of the study ,  n = 12consented to the MRI scan but decided not to during the MRI session,  n = 18 unable to be scheduled,  n = 9 technical problems with scanner,  n = 8 not available on day of scheduled scan  n = 1 baby in NICU. |
| (Merhar et al., 2020) | N=71 (n=42 opioid exposed, n=29 controls) | Opioid exposed: PMA 44.7 (1.2) weeks  Controls: PMA 44.0 (2.0) weeks | 71 | Not reported. |
| (Acosta et al., 2021) | N=105 | 26.1 (7.2) [11–54] days | 105/189 | n=64 failed MRI scanning or motion artifacts in the MR images  n=19 missing genetic data  n=1 missing maternal questionnaire data |
| (Spann et al., 2020b) | N=45 | PMA 42.5 (1.7) weeks | 45/72 | 45 newborns (62.5%) had usable functional and anatomical MRI data. |
| (Graham et al., 2020) | N=34 | 18 (7) [12-37] days | 34/44 | Among them 44 infants having MR imaging, 34 had both valid structural and/or diffusion scans and maternal anxiety scores (2 of these did not have valid depression scores) and were included. |
| (Acosta et al., 2020b) | N=105 | 26.1 (7.2) [11–54] days | 105/189 | n=64 failed MRI scanning or motion artifacts in the MR images  n=19 missing genetic data  n=1 missing maternal questionnaire data |
| (Acosta et al., 2020a) | N=105 | 26.1 (7.2) [11–54] days | 105/189 | n=64 failed MRI scanning or motion artifacts in the MR images  n=19 missing genetic data  n=1 missing maternal questionnaire data |
| (Dowe et al., 2020) | N=97 | 32.91 (6.03) days | 97/149 | n=46 due to motion artifacts or unsuccessful scan  n=6 with missing attention data |
| (Fenchel et al., 2020) | N=241 | PMA 40.92 (1.58) [37.43–44.71] weeks | 241/383 | 383 subjects had undergone successfully structural and diffusion acquisitions, reconstruction, and early preprocessing.  Subjects were excluded due to:  n=87 failed structural or diffusion pipelines or who missing data (e.g., T1 image)  n=17 did not pass diffusion QC (subjects at the lower 5% of motion parameters)  n=46 did not pass diffusion QC (brains moved out of the field of view during scanning, leading to slices missing on the superior surface of the brain)  n=1 with major basal ganglia lesion |
| (Gale-Grant et al., 2020) | N=275 | 40.9 [39.7–42.0] weeks | 275 | Not reported. |
| (Alexander et al., 2020) | N=10 | GA 41.71 (1.31) [40.29–43.00] weeks | 10 | Not reported. |
| (Adibpour et al., 2020) | N=22 | 5.9–22.4 weeks | 21/22 Anatomical T2-weighted  22/22 DTI | Anatomical MRI data could not be acquired for one infant. |
| (Spann et al., 2020a) | N=37 | PMA 42 (1.9) weeks | 37 | Not reported. |
| (Ong et al., 2019) | N=184 | 5-17 days | good T2‐weighted MRI data (n = 184) and good DTI data (n = 122) | All 184 neonates had T2‐weighted MRI data, while only 142 neonates had DTI data. Through visual inspection, only 122 neonates had the good DTI data, partially because DTI was last to be acquired and sensitive to head motion. |
| **Cross-sectional studies, participant age 0 – 1 year** | | | | |
| (Adibpour et al., 2018) | N=22 | 13.8 (4.2) weeks | 22/24 | n=2 due artifacted MRI images |
| (Lebenberg et al., 2019) | N=17 | 3-21 weeks (maturational age, corrected for gestational age at birth, with a reference gestational period of 41 weeks) | 17 | Not reported. |
| (Sethna et al., 2016) | N=39 | 3–6 months, 4.83 (1.15) months | 39/43 | n=3 due to motion artifacts  n=1 due to brain anatomical anomaly |
| (Hazlett et al., 2012) | N=134 (n=98 high genetic risk for autism (HR), n=36 low risk for autism (LR)) | 6 months  HR: 6.2 (0.3) months  LR: 6.3 (0.2) months | 318 children attempted the MRI scans, 88% HR and 83% LR were successfully scanned, for a total of 276 scans. 134 scans were used in this study.  134/318 | n=42 with unsuccessful scans  n=142 out of optimal age window |
| (Deniz Can et al., 2013) | N=19 | 7 months, 6.9 (0.5) [6.0-7.8] months | 19/30 | n=2 due to failure to sleep at the imaging center  n=6 waking during transition to the scanning bed or at the onset of the first scan  n=2 motion artifact  n=1 usable MRI data, missing behavioral measures |
| (Langer et al., 2015) | N=32 (n=14 familiar risk for DD (FHD+), n=18 without familiar risk for DD (FHD-)) | FHD+: 6.6–17.6 months, 333 (118) days  FHD-: 5.1–17.6 months, 298 (99) days | 32 | n=9 poor data quality |
| (Travis et al., 2014) | N=17 | 12–19 months, 15.4 (2.3) months | 17^‡^ | n=2 had only 1 T1-image, due to infant moved or woke up during acquisition |
| (Camacho et al., 2020) | N=38 | 6.62 (0.61) months | 38/80 | n=30 failed to sleep on the scanner bed and did not enter the scanner at all  n=9 woke up during acquisition without returning to sleep  n=1 did not provide a complete acquisition (the superior cortex was cut off)  n=2 due to excessive motion |
| **Cross-sectional studies, participant age 0 – 3 years** | | | | |
| (Dean et al., 2014b) | N=162 (n=60 APOE ε4 carriers, n=102 noncarriers) | 2–25 months  carriers: 391 (196) days  noncarriers: 366 (181) days | 162/162 MWF  59/162 GMV | n=81 did not have T1 data, due to infant woke up prior to the completion of the scan  n=22 problems with the analysis |
| (Gao et al., 2013) | N=147 | - Neonates, 23 (12) days - 1 year, 13 (1) months - 2 years, 24 (1) months | - 51 - 50 - 46   Total 147 | Not reported. |
| (Bruchhage et al., 2020) | N=196 (n=109 boys, n=87 girls) | Boys: 24.9 (18.1) months,  Girls: 23.7 (18.3) months | 196/204 | n=8 due to motion after data preprocessing |
| **Cross-sectional studies, participant age 0 – 6 years** | | | | |
| (O’Muircheartaigh et al., 2014) | N=183 | 2–48 months [79–1455] days^†^ | 183 | Not reported. |
| (Deoni et al., 2013) | N=133 (n=85 breastfed, n=38 formula-fed, n=51 a mixture of breast milk and formula)^‡^ | 10–48 months [305–1541] days^†^ | 133 | Not reported. |
| (Dean et al., 2014c) | N=122 (+n=63 independent subsample) | 3–60 months, 690.14 [70-1809] days^†^ | 122 (+63) | Not reported. |
| (Chen et al., 2019) | N=28 | - 0-1 years, 5.6 (3.5) months - 1-2 years, 16.7 (3.5) months - 2-3 years, 27.9 (2.0) months - 3-4 years, 42.3 (5.3) months - 4-5 years, 54.4 (4.9) months | - 7 - 11 - 2 - 3 - 5   Total 28 | Not reported. |
| (Deoni et al., 2015) | N=215 | 1-6 years, 1051 (517) [363-2198] days† | 215 | Not reported. |
| (O’Muircheartaigh et al., 2013) | N=108 | 1-6 years, 3.11 years | 108 | Not reported. |
| **Longitudinal studies, participant age 0-1 year** | | | | |
| (Chang et al., 2016) | N=139 (n=32 tobacco exposed, n=36 methamphetamine/tobacco exposed, n=71 unexposed) | - 1 week - 1–2 months - 2–4 months | 223/325  109 infants completed 1 time point, 72 completed 2 time points, and 44 completed 3 time points.^‡^ | n=102 due to inability to remain asleep, excessive head motion, or different scanning parameters during optimization phase |
| (Qiu et al., 2013b) | N=175 | - 5-17 days, PMA 40.1 (1.2) weeks - 6 months, PMA 66.4 (1.9) weeks | - 175/189 - 35/42   210/231 (n=140 scanned once, n=35 twice,) | n=5 due to demographic reasons  n=9 motion in the MRI  n=7 invalid data |
| (Kim et al., 2016) | N=33 | - 2 weeks, 26 (8) days - 3 months, 102 (25) days - 6 months, 189 (9) days - 9 months, 279 (15) days - 12 months, 372 (14) days | - 33 - 33 - 33 - 33 - 33   Total 165 (All subjects scanned five times.) | Not reported. |
| (Meng et al., 2017) | N=15 | - 1 month - 3 months - 6 months - 9 months - 12 months | - 15 - 15 - 15 - 15 - 15   Total 75 (All subjects scanned five times.) | Not reported. |
| (Wang et al., 2012) | N=28 | - neonates - 3 months - 6 months - 9 months - 12 months (or older) | - 28 - 28 - 28 - 28 - 28   Total 140 (All subjects scanned five times.) | Not reported. |
| (Choe et al., 2013) | N=27 | - 3–4 months - 6–7 months - 12–13 months | - 5 - 19 - 9   Total 33 (n=21 scanned once, n=6 twice) | Not reported. |
| (Li et al., 2014b) | N=13 | - 0 months - 3 months - 6 months - 9 months - 12 months - 18 months | - 13 - 13 - 13 - 13 - 13 - 13   Total 78 (All subjects scanned seven times.) | Not reported. |
| **Longitudinal studies, participant age 0-3 years** | | | | |
| (Hu et al., 2019) | N=50 | - 1 month, 27.3 (9.0) [14-48] days - 3 months, 94.6 (8.6) [81-116] days - 6 months, 190.0 (12.8) [169-225] days - 9 months, 278.1 (13.9) [251-309] days - 12 months, 375.5 (14.5) [352-418] days - 18 months, 556.8 (19.8) [507-613] days - 24 months, 738.2 (27.3) [666-797] days | - 39 - 36 - 41 - 36 - 36 - 40 - 23   Total 251 | Not reported. |
| (Swanson et al., 2015) | N=77 | - 6 months, 6.71 (0.60) months - 12 months, 12.73 (0.72) months - 24 months, 24.63 (0.83) months | - 60 - 63 - 45 or 46^‡^   (n=16 scanned once, n=30 twice, n=31 three times) | Not reported. |
| (Li et al., 2015a) | N=73 | - Neonatal, 0.85 (0.36) [0.3-2.0] months - 1 year, 1.08 (0.06) [0.94-1.18] years - 2 years, 2.08 (0.10) [1.81-2.40] years | - 73 - 73 - 73   Total 219 (All subjects scanned three times.) | Not reported. |
| (Li et al., 2015b) | N=35 | - 1 months - 3 months - 6 months - 9 months - 12 months - 18 months - 24 months | - 35 - 28 - 31 - 27 - 29 - 31 - 21   Total 202 (Each infant scanned 4 to 7 times, 5.8 on average.) | Not reported. |
| (Li et al., 2014c) | N=73 | - Neonates, 25.5 (10.8) days - 1 year, 392.8 (22.1) days - 2 years, 758.1 (38.1) days | - 73 (90%) - 73 (66%) - 73 (60%)   Total 219 (All subjects scanned three times.) | Subjects were part of a large prospective study witch success rate for usable MRI scans were: 90% for neonates, 66% for 1-year-old and 60% for 2-year-old. |
| (Li et al., 2014a) | N=73 | - Neonates, 25.5 (10.8) days - 1 year, 392.8 (22.1) days - 2 years, 758.1 (38.1) days | - 73 - 73 - 73   Total 219 (All subjects scanned three times) | Not reported. |
| (Li et al., 2013) | N=73 | - Neonates, 25.5 (10.8) days - 1 year, 392.8 (22.1) days - 2 years, 758.1 (38.1) days | - 73 - 73 - 73   Total 219 (All subjects scanned three times) | Not reported. |
| (Alcauter et al., 2015) | N=86 | - Neonates, 30.25 (17) days - 1 year, 397 (32) days - 2 years, 758 (25) days | - 36/111 - 45/118 - 31/78   112/307 | 307 scans were retrospectively identified (n=111 neonates, n=118 1-year-olds and n=78 one-year-olds), and final sample included 112 scans. Scans were excluded during preprocessing. |
| (Alcauter et al., 2014) | N=143 | - Neonates, 33 (19) days - 1 year, 397 (35) days - 2 years, 762 (33) days | - 112 - 129 - 92   Total 333 (n=96 scanned twice, n=47 three times) | Not reported. |
| (Gao et al., 2014b) | N=288 | - 1 month, 37 (18) days - 12 months, 397 (26) days - 24 months, 765 (32) days | - 178 - 132 - 100   Total 410 | Not reported. |
| (Gao et al., 2014a) | N=143 | - Neonates, 33 (19) days - 1 year, 13.3 (1.2) months - 2 years, 25.4 (1.1) months | - 112 - 129 - 92   Total 333 (n=96 scanned twice, n=47 three times) | Not reported. |
| (Sadeghi et al., 2013) | N=26 | - neonates - 1 year - 2 years | - 23 - 22 - 14   Total 59 (n=2 scanned once, n=15 twice, n=9 three times) | n=4 sub-optimal scans |
| (Geng et al., 2016) | N=118 | - Neonates, GA 42.5 (1.5) weeks - 1 year, GA 94.1 (3.5) weeks - 2 years, GA 145.5 (3.2) weeks | - 115 or 118 ^‡^ - 100 - 82   Total 300. | Not reported. |
| (Geng et al., 2012) | N=211 | - neonates: 3.02 (2.00) weeks - 1 year: 54.60 (3.92) weeks - 2 years: 106.25 (4.36) weeks | - 163 - 77 - 55   Total 295 (n=141 scanned once, n=56 twice, n=14 three times) | Not reported. |
| (Bompard et al., 2014) | N=24 | 0-27 months  Every 3 months during the first year.  Every 6 months during the second year. | 137 left LV/168  133 right LV/168  (n=7 scanned four times, n=7 five times, n=7 six times, n=3 seven times) | n=7 missing appointments  n=4 subject attrition  n=1 motion  n=18 out of target age  n=5 failure of SPHARM-PDM |
| (Schmied et al., 2020) | N=104 | - 6.7 (0.7) [5.5–9.0] months - 12.7 (0.7) [11.8–15.9] months - 24.8 (0.9) [23.6–28] months | - 87 - 81 - 56   Total 224 | Not reported. |
| (Wang et al., 2019) | N=43 | - 1 months [0.47-1.60] - 3 months [2.73-3.87] - 6 months [5.73-7.50] - 9 months [8.73-10.13] - 12 months [11.73-13.93] - 18 months [16.90-20.43] - 24 months [22.20~26.57] | - 33 - 31 - 32 - 30 - 31 - 33 - 20   Total 210 | Not reported. |
| (Ahn et al., 2019) | N=240 (n=202 healthy control, n=38 high risk) | Healthy controls:   - neonates 22.52 (11.41) days - 1 year 382.9 (22.61) days - 2 year 745.3 (25.01) days   High risk for schizophrenia:   - neonates 32.68 (16.31) days - 1 year 390.3 (31.71) days - 2 year 766.8 (42.00) days | - 161 - 119 - 92   Total 372 | Not reported. |
| **Longitudinal studies, participant age 0-6 years** | | | | |
| (Dai et al., 2019a) | N=210 | 2-48 months, [65-1481] days^†^ | Total 416 (n=93 were scanned once, n=60 twice, n=30 three times, n=23 four times, n=3 five times, n=1 six times) | n=22 due to motion-related artifacts |
| (Dai et al., 2019b) | N=222 | 2-48 months, [65-1489] days | Total 445 (1-6 scans per child, median 2) | Not reported. |
| (Dean et al., 2015a) | N=209 | 3–48 months [76-1526] days^†^ at recruitment | 327  (n=128 scanned once, n=58 twice, n=21 three times, n=5 four times) | Not reported. |
| (Dean et al., 2014a) | N=220 | 3–48 months | Total 384 (164 subjects were scanned at least twice).  Success rate   - of scanning during the first time visit near 90% - of scanning during second or third visit 100% - acquiring usable MRI 96% - overall 97% | n=9 due to image artifacts (first time scans) |
| (Chen et al., 2014) | N=29 | - neonates 0.07 (0.07) years - 1 year. 1.05 (0.05) years - 2 years, 2.03 (0.07) years - 4 years, 4.15 (0.16) years | - 25 - 16 - 23 - 7   Total 71 (All subjects were scanned at least twice.) | Not reported. |
| (Deoni et al., 2016) | N=257 | 3 months - 5 years, [98–1814] days^†^ | 257 were scanned at least once.  A subset of 126 children received additional longitudinal scans: n=126 were scanned twice, n=39 at least three times, n=15 at least four times, and n=4 five times. | Not reported. |
| (Dean et al., 2015b) | N=108 | 2.5 months – 5.5 years [70–1928] days^†^ at recruitment  Subjects under 2 years of age were scanned every 6 months, over 2 years of age were scanned yearly. | 260 (n=73 scanned twice, n=26 three times, n=9 four times) | Not reported. |
| (Croteau-Chonka et al., 2016) | N=134 | 1–6 years, 1044 (523) [363-2198] days^†^ | 177  (n=98 scanned once, n=29 twice, n=7 three times) | Not reported. |
| (Remer et al., 2020) | N=233 (n=74 including ε4 carriers, n=149 non-carriers) | 2 months to 5.7 years  Mean time between repeat scans was 247 days for participants under 2 years of age and 401 days for participants over 2 years of age. | all had at least one MRI;  115 had at least two MRIs;  41 had at least 3 MRIs;  16 had at least four MRIs.  Total 403 scans. | Not reported. |

Abbreviations: SD=standard deviation, SE=standard error, DD=developmental dyslexia, LV=lateral ventricle, PMA=postmenstrual age, DOT=diffuse optical tomography, SPHARM-PDM=spherical harmonic based point distribution model, GA=gestational age, APOE=apolipoprotein E, MWF=myelin water fraction, GMV=gray matter volume, ^†^corrected to a 40-week gestation, ^‡^unreliable information, reported as is the article
